# A Review of Neuroprotective Effects and Mechanisms of Ginsenosides From Panax Ginseng in Treating Ischemic Stroke

**DOI:** 10.3389/fphar.2022.946752

**Published:** 2022-07-07

**Authors:** Aimei Zhao, Nan Liu, Mingjiang Yao, Yehao Zhang, Zengyu Yao, Yujing Feng, Jianxun Liu, Guoping Zhou

**Affiliations:** ^1^ Department of Acupuncture and Moxibustion, Neuroscience Centre, Integrated Hospital of Traditional Chinese Medicine, Southern Medical University, Guangzhou, China; ^2^ Beijing Increasepharm Safety and Efficacy Co., Ltd., Beijing, China; ^3^ Beijing Key Laboratory of Pharmacology of Chinese Materia Region, Institute of Basic Medical Sciences, Xiyuan Hospital of China Academy of Chinese Medical Sciences, Beijing, China; ^4^ Department of Anesthesiology, Punan Hospital, Shanghai, China

**Keywords:** panax ginseng, ginsenosides, traditional Chinese medicine, cerebral ischemic stroke, neuroprotection mechanisms

## Abstract

Ischemic stroke has been considered one of the leading causes of mortality and disability worldwide, associated with a series of complex pathophysiological processes. However, effective therapeutic methods for ischemic stroke are still limited. Panax ginseng, a valuable traditional Chinese medicine, has been long used in eastern countries for various diseases. Ginsenosides, the main active ingredient of Panax ginseng, has demonstrated neuroprotective effects on ischemic stroke injury during the last decade. In this article, we summarized the pathophysiology of ischemic stroke and reviewed the literature on ginsenosides studies in preclinical and clinical ischemic stroke. Available findings showed that both major ginsenosides and minor ginsenosides (such as Rg3, Rg5, and Rh2) has a potential neuroprotective effect, mainly through attenuating the excitotoxicity, Ca^2+^ overload, mitochondria dysfunction, blood-brain barrier (BBB) permeability, anti-inflammation, anti-oxidative, anti-apoptosis, anti-pyroptosis, anti-autophagy, improving angiogenesis, and neurogenesis. Therefore, this review brings a current understanding of the mechanisms of ginsenosides in the treatment of ischemic stroke. Further studies, especially in clinical trials, will be important to confirm the clinical value of ginseng and ginsenosides.

## Introduction

Stroke is one of the leading causes of disability and mortality worldwide, which creates a significant economic burden on the healthcare system (Johnson et al., 2019). Ischemic stroke (IS) is the primary stroke subtype, accounting for approximately 87% of stroke cases (Virani et al., 2020). The middle cerebral artery (MCA) is the most commonly affected vascular territory in cerebral ischemic stroke (Navarro-Orozco and Sánchez-Manso, 2022), and an intravascular blood clot or thrombus usually causes vascular occlusion. Clinically, thrombolytic therapy and thrombectomy are the only approved methods for treating acute ischemic stroke (Campbell and Khatri, 2020), restoring blood flow to the ischemic brain and rescuing damaged neurons in the ischemic penumbra. However, these treatments have limited time windows, intravenous alteplase (rtPA) restricted within 4.5 h, endovascular thrombectomy within 24 h (Lees et al., 2010; Powers et al., 2019), to reduce the risk of hemorrhagic transformation, and only a minority of patients benefit from the treatments timely. Therefore, exploring new drugs or therapies is necessary to prolong the therapeutic window and improve patient outcomes.

In ischemic stroke, the obstruction of brain blood vessels deprives the essential nutrients and oxygen to brain cells, causing a sudden onset of neurological deficit. The ischemic insult may lead to irreversible damage or death to neurons in the ischemic core, while the neurons in the penumbral area surrounding the ischemic core may be salvageable with effective brain-protective treatments. Ischemic stroke involves a variety of mechanisms, such as excitotoxicity, mitochondrial dysfunction, oxidative stress, inflammation, autophagy, and blood-brain barrier (BBB) damage (George and Steinberg, 2015; Chamorro et al., 2016; Wang P. et al., 2018). Mitochondrial dysfunction occurs within minutes of ischemic stroke, resulting in depletion of adenosine triphosphate (ATP) and membrane depolarization, followed by sustained glutamate release and intracellular Ca^2+^ overload. The increased intracellular calcium leads to the overproduction of reactive oxygen species (ROS) and activates inflammatory responses, triggering the death of damaged neurons and the leakage of BBB (Zhou et al., 2018). These pathophysiological mechanisms overlap and correlate with the development of ischemic stroke and are potential pharmacological targets for treating ischemic stroke.

As a traditional herbal medicine, Panax ginseng has been widely used in treating and preventing diseases for thousands of years in East Asian countries, especially in China, Korea, and Japan. The botanical name “Panax” implies “all-healing” in Greek, which stemmed from the traditional belief that ginseng has healing properties in all aspects of the body (Kim, 2018). Among the eleven ginseng species, Panax ginseng (Asian or Korean ginseng), Panax quinquefolius (North American ginseng), and Panax notoginseng are three particularly important for medicinal use (Wang et al., 2020). Panax ginseng contains various pharmacological components, such as ginsenosides, polysaccharides, and polyphenols (Zheng et al., 2017). Ginsenosides are considered the main active ingredients of Panax ginseng, Panax quinquefolius, and Panax notoginseng for the pharmaceutical functions, which are mainly accumulated in roots, stems, leaves, flowers buds, and berries (Kim et al., 2018). About 200 ginsenosides have been identified from ginseng, including major ginsenosides (Rd, Rb1, Rb2, Rc, Re, Rg1, etc.) and minor ginsenosides (Rh1, Rh2, Rg3, Rg5, etc.) (Hyun et al., 2022). According to the chemical structures, ginsenosides can be divided into protopanaxadiol (PPD), protopanaxatriol (PPT), and oleanolic acid. PPD mainly includes ginsenosides Rd, Rb1, Rb2, Rb3, Rg3, Rg5, Rh2, F2, and compound K. PPT includes the ginsenosides Rg1, Rg2, Re, Rf, Rh1, and F1, while the typical representative ginsenoside of the oleanolic acid is ginsenoside Ro (Lu et al., 2022). As a natural product, ginseng has a wide range of pharmacological effects, such as anti-oxidative and anti-cancer, enhancing immunity, energy, and sexuality, and combating neurological diseases, diabetes mellitus, and cardiovascular diseases (Ratan et al., 2021). Currently, growing evidence shows that ginsenosides have neuroprotective effects *in vivo* and *in vitro* and have excellent potential as novel candidate agents for ischemic stroke. It can be used to treat ischemic stroke *via* reducing neurotoxicity (Zhang C. et al., 2020), anti-oxidant (Chu et al., 2019), anti-inflammation (Zhu et al., 2012), anti-apoptosis (Li et al., 2010), anti-autophagy (Huang et al., 2020), regulating blood-brain barrier permeability (Zhang X. et al., 2020), promoting angiogenesis (Chen J. et al., 2019) and neurogenesis (Gao et al., 2010) to alleviate nerve damage and promote nerve repair.

This article reviews the literature on treating ischemic stroke with ginsenosides, including preclinical and clinical experimental studies. Studies of ginsenosides in treating cerebral ischemia published until March 2022 were identified from the PubMed database. We summarized the pathophysiologies of cerebral ischemia stroke and the potential mechanisms of ginsenosides in treating ischemic stroke. Our work brings a current understanding of the mechanisms of ginsenosides in the treatment of ischemic stroke.

## Pathophysiologies of Ischemic Stroke

### Excitotoxicity

Excitotoxicity is one of the significant events in cerebral ischemia, playing a key role in neuronal death (Rothman and Olney, 1986). After cerebral ischemia, rapid and massive release and uptake inhibition of the excitatory amino acid glutamate leads to energy failure (Chamorro et al., 2016). The function of ion pumps is required with ATP to transform the sodium (Na^+^), potassium (K^+^), and Ca^2+^ between intracellular and extracellular. With ATP depletion, the Ca^2+^ cannot be pumped out of neuron cells and causes glutamate release (Luoma et al., 2011). Postsynaptic receptors of glutamate include ionotropic receptors or metabotropic receptors (mGluRs), the ionotropic type receptor, NMDA (N-methyl-D-aspartate) receptor, which primarily regulates the excitotoxic response (Kaplan-Arabaci et al., 2022). Overactivation of glutamate receptors leads to the opening of receptor-gated calcium channels and Ca^2+^ influx, and the increase of intracellular Ca^2+^ causes a series of pathological reactions in the cytoplasm and nucleus (Lai et al., 2014). Moreover, Ca^2+^ overload in mitochondrial activates the downstream apoptotic pathway, inducing mitochondrial destruction and cell apoptosis (Szydlowska and Tymianski, 2010). Astrocyte glutamate transporter excitatory amino-acid transporter 2 (EAAT2 or GLT-1) is the primary glutamate transporter in the brain, playing a pivotal role in sustaining glutamate homeostasis (Tzingounis and Wadiche, 2007). Therefore, regulating the excitatory neurotransmitter glutamate and Ca^2+^ influx significantly reduces the excitotoxicity after cerebral ischemia.

### Inflammation

Inflammatory response plays a crucial role in ischemic stroke pathogenesis, which contributes to all the stages of ischemic stroke (Drieu et al., 2018). Inflammatory response at the blood-endothelial interface, including adhesion molecules, cytokines, chemokines, and leukocytes, is an essential cerebral infarction tissue injury mechanism (Zhu et al., 2022). Astrocytes and microglia are the primary cells in the brain that mediate inflammatory responses in response to ischemic brain injury (Mo et al., 2020). Astrocyte hypertrophy and proliferation are extensive responses to neuronal injury. Stroke-induced brain injury activates microglia polarization into pro-inflammatory, classical (M1) or anti-inflammatory, alternative (M2) phenotypes (Song et al., 2019). M1 microglia produce large amounts of pro-inflammatory mediators, such as tumor necrosis factor *α* (TNF*α*), interleukin (IL)-1*β*, IL-6, interferon-*γ* (IFN-*γ*), inducible nitric oxide synthase (iNOS), and proteolytic enzymes (Yenari et al., 2010). While M2 microglia is characterized by the effects of pro-angiogenic and anti-inflammatory, producing IL-4, IL-10, transforming growth factor *β* (TGF-*β*), and vascular endothelial growth factor (VEGF) (Qin et al., 2019). Inflammation after cerebral ischemia with contrasting effects, as it can promote nerve repair as well as aggravate secondary brain damage. Toll-like receptors (TLRs), nuclear factor-kappa B (NF-κB), and mitogen-activated protein kinases (MAPK) signaling pathways are related to the activation of inflammation in ischemic stroke (Mo et al., 2020). TLRs are transmembrane proteins expressed in microglia, astrocytes, neurons, and cerebral endothelium (Marsh et al., 2009), which can induce inflammatory responses by regulating cytokine and chemokine production. NF-κB participates in transcriptional induction of pro-inflammatory genes, such as cell adhesion molecules, cytokines, matrix metalloproteinases (MMP), and growth factors. p38 MAPK plays a vital role in inflammation-mediated ischemic injury (Sun and Nan, 2016). In addition, the NOD-like receptor (NLR) family, pyrin domain containing 3(NLRP3) inflammasome can detect tissue damage and pathogen invasion through innate immune cell sensor components commonly known as pattern recognition receptors (PRRs). PRRs promote activation NF-κB and MAPK pathways, thus increasing the transcription of protein-coding genes associated with NLRP3 (Xu et al., 2021).

### Oxidative Stress

Oxidative and nitrosative stress present a challenge to ischemic stroke, which is caused by the excessive production of reactive oxygen species (ROS) and reactive nitrogen species (RNS) (Allen and Bayraktutan, 2009). Excessive ROS can result in lipid peroxidation and damage proteins and DNA, initiating a cascade of deleterious cellular processes that promote cell death. It often results from ROS/RNS production and antioxidant systems imbalance. Under physiological conditions, ROS and RNS can be scavenged by endogenous antioxidant enzymes or non-enzyme, including superoxide dismutase (SOD), catalase (CAT), glutathione peroxidase (GPX), glutathione-S-transferase (GST), and glutathione (GSH) (Tang D. et al., 2019). After cerebral ischemia, ROS and RNS have been shown in phagocytes, vascular cells, and glial cells in the penumbra. ROS is composed of superoxide anions (O_2_^−^), hydrogen peroxide, hydroxyl radical, and hydroperoxyl radicals, a by-product of oxygen metabolism in mitochondria. RNS mainly includes nitric oxide (NO) and peroxynitrite anion (ONOO^−^), while ONOO^−^ is formed by the rapid reaction of NO and O_2_^−^ (He et al., 2021). Nuclear factor erythroid 2-related factor (Nrf2) is a transcription factor that regulates the expression of endogenous antioxidant enzymes, and Nrf2/ARE is an important endogenous anti-oxidative stress signaling pathway (Wu et al., 2020). Actively protecting mitochondrial function, antioxidation, free radical scavenging, and slowing down oxidative stress have become effective strategies in saving neurons from the pathological processes of cerebral ischemia-reperfusion. Anti-oxidative stress, scavenging free radicals, and protecting mitochondrial function have become effective strategies to save neurons from the pathological process of ischemic injury.

### Apoptosis/Pyroptosis/Ferroptosis

Multiple cell death pathways are implicated in the pathogenesis of ischemic stroke (Tuo et al., 2022). Apoptosis is a typical form of programmed or regulated cell death. Recent studies have revealed novel programmed or regulated cell death types, including pyroptosis and ferroptosis (Galluzzi et al., 2018).

Apoptosis can be triggered through either the intrinsic or the extrinsic pathway. The initial morphological changes in apoptosis have been observed in post-ischemic stroke neurons, which involve cell shrinkage and cytoplasmatic condensation, nuclear membrane breakdown, and formation of apoptotic bodies (Linnik et al., 1993). The intrinsic signaling cascade of apoptosis can be mediated by calpain, ROS, and DNA damage. Excessive accumulation of Ca^2+^ and ROS in intracellular triggers activation of calpains and one of the substrates-B-cell leukemia/lymphoma 2 (Bcl-2). Bcl-2 is an anti-apoptotic protein that could interact with Bax on the mitochondrial membrane, causing a release of various proapoptotic factors, including cytochrome C (Cytc) and apoptosis-inducing factor (AIF) (Chao and Korsmeyer, 1998). Cystic complexes form an apoptosome with apoptotic protein-activating factor-1 and procaspase-9, activating caspase-3 and initiating cell death (Wang et al., 2019). ROS can damage the plasma membrane and DNA; DNA damage activates the nuclear pathway of cell death through the phosphorylation of p53 or translocation of nucleophosmin (Culmsee and Krieglstein, 2007). The extrinsic apoptosis pathway is triggered by the extracellular death ligands (TRAIL, FasL, TNF-*α*) that bind to death receptors (TRAILR, Fas, TNFR1) and Fas-associated death domain (FADD), creating a death-inducing signaling complex with procaspase-8. Activated caspase-8 activates downstream effector caspases (such as caspase-3) by direct proteolytic cleavage (Muhammad et al., 2018; Tuo et al., 2022).

Pyroptosis is an inflammatory form of programmed cell death that inflammasome activation can cause. The inflammasome is a protein complex that can be activated by infection, metabolic imbalances, and tissue injury (Broz and Dixit, 2016). Several inflammasome sensor proteins have been identified, including the NLRP1, NLRP3, NLRP4, and absent in melanoma 2 (AIM2), which trigger the downstream inflammatory response (Fann et al., 2013a). Inflammasomes, including canonical and noncanonical types, canonical inflammasomes like the NLRP3 activate caspase-1, whereas noncanonical inflammasomes activate mouse caspase-11 or human caspase-4 and caspase-5 (Hu J. J. et al., 2020). Gasdermin D (GSDMD) is the key effector of pyroptosis, downstream of inflammasome pathways, and a substrate for inflammatory caspases-1,4, 5, and 11 (Liu Z. et al., 2019). Caspase-1 or caspase-11 can cleave GSDMD into an N-terminal fragment (GSDMD-N) and C-terminal product (GSDMD-C) (Shi et al., 2015). Once caspase-1 is activated, pro-IL-1*β* and pro-IL18 can be divided into biologically active, mature, pro-inflammatory cytokines released into the extracellular environment, causing neuronal cell toxicity (Tuo et al., 2022). Inhibition or knockout of caspase-1 is neuroprotective in focal stroke models (Fann et al., 2013b).

Ferroptosis is an iron-dependent form of regulated cell death (Dixon et al., 2012), with iron accumulation and lipid peroxidation. Excessive intracellular iron accumulation elevates ROS by Fenton reaction, leading to ferroptosis cell death by irresistible lipid peroxidation. Studies have shown that iron deposition, lipid peroxidation, and neuronal death in the brain were significantly increased in an adult rat model of ischemic stroke (Kondo et al., 1997; Park U. J. et al., 2011). Glutathione peroxidase 4 (GPX4) plays an important role in suppressing ferroptosis, which functions to reduce lipid peroxides in cellular membranes. GPX4 uses GSH to eliminate the production of phospholipid hydroperoxides (PLOOH), the primary mediator of chain reactions in lipoxygenases (Tang D. L. et al., 2019). GSH is the most abundant antioxidant in the cell, synthesized from glutamate, cysteine, and glycine, among which cysteine is the rate-limiting precursor (Lee et al., 2020). The intracellular cysteine level mainly depends on extracellular cystine uptake by system Xc^−^ (Koppula et al., 2018), which consists of a regulatory subunit solute carrier family 3 member 2 (SLC3A2) and a catalytic subunit solute carrier family 7 member 11 (SLC7A11). Correspondingly, the inactivation of GPX4 or SLC7A11 induces ferroptosis. The levels of GPx4 and SLC7A11 were found to be decreased in MCAO rats compared with those in the sham group (Lan et al., 2020).

### Autophagy

Autophagy-dependent death, known as type 2 programmed cell death (Shen et al., 2013), plays a vital role in maintaining cellular homeostasis after cerebral ischemia. Ischemia and hypoxia cause cell dysfunction of energy metabolism, leading to the destruction of the cytoskeleton and loss of homeostasis (Mo et al., 2020). Autophagy is initiated by nucleating a double membrane, which elongates into an autophagosomal vesicle that encapsulates damaged macromolecules and organelles (Klionsky et al., 2016). A cascade of autophagy-related proteins (ATGs) plays critical roles in autophagic membrane dynamics and processes (Liu and Levine, 2015). LC3-II is a biological marker of autophagosome formation localized to the autophagosome membrane. Mammalian target of rapamycin (mTOR) is one of the critical targets for autophagy regulation, a serine/threonine-protein kinase that belongs to the phosphatidylinositol 3-kinase (PI3K) related kinase family (Glick et al., 2010). Typical autophagy is triggered through a core pre-activation complex composed of ULK1/2, ATG13, and FIP200 proteins. AMPK is a central regulator of metabolism and autophagy and can phosphorylate ULK1 to activate autophagy (Jia et al., 2020), a potential therapeutic target for ischemic stroke (Jiang et al., 2018). In moderate hypoxia, hypoxia-inducible factor-1*α* (HIF-1*α*) regulates autophagy through upregulating expression of Bcl-2 and 19-kDa interacting protein 3 (BNIP3), while BNIP3 mediates autophagy by disrupting the interaction of Beclin-1 with Bcl-2 (Matsui et al., 2008). A report showed that knockdown of Beclin-1 can prevent secondary neurodegenerative damage after focal cerebral infarction by inhibiting autophagy activation (Xing et al., 2012).

### Others

Cerebral ischemia initiates a complex cascade of pathophysiological events. In addition to the pathophysiologic reviewed above, BBB permeability, angiogenesis, and neurogenesis are crucial mechanisms for cerebral ischemia and reperfusion. The BBB is a cellular barrier composed of tight junctions between vascular endothelial cells interfaced with pericytes and astrocytes (Singh et al., 2016), which protects the central nervous system (CNS) by regulating the transport of substances between the blood and brain. Inflammatory cytokines, such as TNF-*α* and IL-1*β*, can increase the permeability of BBB to entrance into the CNS (Smith et al., 2016). The increased biphasic permeability of BBB leads to cerebral angiogenic edema, hemorrhage, and mortality during ischemic stroke-reperfusion (Knowland et al., 2014). Angiogenesis involves sprouting new vessels from existing vessels, predominantly induced by vascular endothelial growth factor (VEGF) (Ferrara and Adamis, 2016). It is critical to repair tissue regeneration under wound healing, hypoxia, and chronic ischemia (Fan et al., 2018). Under hypoxic conditions, HIF-1*α* plays a crucial role in pathophysiological angiogenesis by directly regulating VEGF, and HIF-1*α*/VEGF may be an important pathway for the regulation of angiogenesis (Hu Q. et al., 2020). Neurogenesis is a complex process that generates new functional neurons and glial cells from neural stem cells (NSCs), mainly in the subventricular zone (SVZ) and the subgranular zone of the dentate gyrus (DG) of the hippocampus, involving proliferation, differentiation, migration, and maturation (Nada et al., 2014). Accumulative evidence supports that newborn neurons have critical physiological functions in neuroplasticity, learning and memory, and emotion regulation (Berg et al., 2019). After cerebral ischemia, the increased expression of brain-derived neurotrophic factor (BDNF), platelet-derived growth factor-B (PDGF-B), transforming growth factor-beta (TGF-*β*), fibroblast growth factor 2 (FGF2), and VEGF, may promote both angiogenesis and axonal outgrowth (Hatakeyama et al., 2020). Therefore, activation of endogenous neurogenesis plays a vital role in promoting neurological function recovery.

## Neuroprotective Effects of Ginseng and Ginsenosides in Ischemic Stroke

**FIGURE 1:**
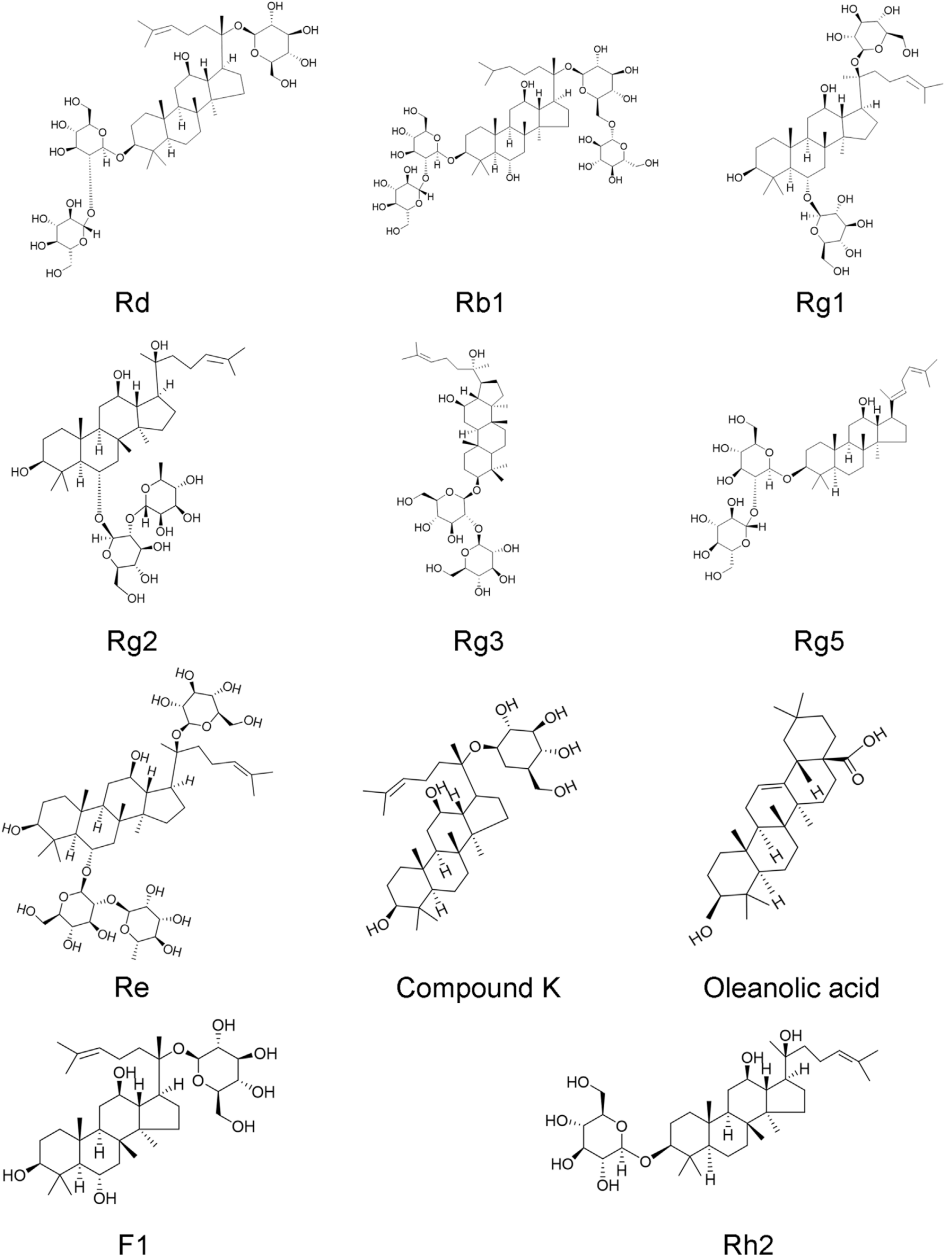
Chemical structures of ginsenoside Rd, Rb1, Rg1, Rg2, Rg3, Rg5, Re, Rh2, F1, compound K, and oleanolic acid.

Ginseng, the root of Panax ginseng, has been widely used to treat cerebrovascular diseases in Asian countries. Ginsenosides are the major bioactive components of ginseng, responsible for its pharmacological activities (Kim et al., 2020). Now, accumulated studies show that ginseng and ginsenosides have many positive effects on treating and preventing cerebral ischemic stroke. Ginsenosides with neuroprotective effects mainly include ginsenoside Rb1 (Liu A. et al., 2018), ginsenoside Rd (Zhang X. et al., 2020), ginsenoside Re (Chen et al., 2008), ginsenoside Rg1 (Zheng et al., 2019), ginsenoside Rg2 (Zhang G. et al., 2008), ginsenoside Rg3 (He et al., 2017), ginsenoside Rg5 (Cheng et al., 2019), ginsenoside Rh2 (Bae et al., 2006), ginsenoside F1 (Zhang et al., 2019), Compound K (Huang et al., 2020), Oleanolic acid (Lin et al., 2021) (Figure 1). Overall, the neuroprotective effects of ginseng and ginsenosides against cerebral ischemia are mediated by the regulation of excitotoxicity, Ca^2+^ overload, inflammation, mitochondria dysfunction, oxidative stress, apoptosis, pyroptosis, autophagy, BBB permeability, angiogenesis, and neurogenesis, as shown in Table 1 and Table 2. The content of ginsenosides in Panax ginseng is also shown in Table 1.

**TABLE 1:** Summary of effects and mechanisms of ginseng and ginsenosides *in vitro* and *in vivo* models.

Gensing and gensinosides	Content of ginsenosides in panax ginseng	Animals/Cells and Dosage	Model	Mechanisms	Effects	References
KRG		C57BL/6 mice, 100 mg/kg	HI	Nrf2↑	Antioxidant	Liu et al. (2020)
				AQP4↓		
KRG		C57BL/6 mice, 100 mg/kg	HI	NQO1, HO1, SOD2, Gpx1, IL-10↑	Antioxidant, anti-inflammation	Liu et al. (2019b)
				IL-1*β*, iNOS↓		
KRG		C57BL/6 mice, 100 mg/kg	pdMCAO	Nrf2↑	Oxidative stress, inflammation, improve long-term recovery	Liu et al. (2019a)
				AQP4↓		
KRG		C57BL/6 mice, 100 mg/kg	pdMCAO	NQO1, HO1, SOD2, Gpx1↑	Antioxidant, attenuate acute sensorimotor deficits, improve long-term functional recovery	Liu et al. (2018b)
				Nrf2 pathway		
RGE		C57BL/6 mice, 360 mg/kg	MCAO	ASK1, ROS, TUNEL↓	Oxidative stress, apoptosis	Cheon et al. (2013)
KRG		SD rats, 100 mg/kg	MCAO/R	MDA↑	Antioxidant	Ban et al. (2012)
				GPx, SOD, CAT↓		
BG		SD rats, 100 or 400 mg/kg	MCAO	Cholinergic immunoreactivity, NADPH-d↑	Improve learning and memory	Park et al. (2011a)
KRG		SD rats, 100 mg/kg	tMCAO	TNF-*α*, IL-1*β*, IL-6↓	Inflammation	Lee et al. (2011)
PGE		Wistar rats, 200 mg/kg	TGCI	SOD, GPx↑	Antioxidant	Kim et al. (2009)
				MDA↓		
KGT		Swiss albino rat, 350 mg/kg	MCAO	GSH, GR, CAT, GST, GPx, SOD↑	Antioxidant	Shah et al. (2005)
				LPO↓		
GTS		Wistar rats, 25 mg/kg	MCAO	BrdU+/NeuN+↑	Neurogenesis	Zheng et al. (2011)
Rd	0.07 ± 0.03%Park et al. (2013)	C57BL/6 mice, 10, 20, 40 mg/kg	MCAO/R	miR-139-5p, Nrf2↑	Pyroptosis	Yao et al. (2022)
	0.07–0.19%Chen et al. (2019b)	Cortical neuron, 5, 10, 20 μM	OGD/R	NLRP3, ASC, Caspase 1 p20, and GSDMD-N, FoxO1, Keap1, ROS, TXNIP↓ miR-139-5p/FoxO1/Keap1/Nrf2 axis		
Rd		SD rats, 30 mg/kg	MCAO	NF-κB, MMP-9↓NF-κB/MMP-9 pathway	BBB inflammation	Zhang et al. (2020b)
Rd		SD rats, 10 mg/kg	MCAO	P-NR2b at Ser-1303, calcineurin↓	Excitotoxicity	Zhang et al. (2020a)
		Cortical neurons, 0 μM, 3 μM, 10 μM, 100 μM	OGD			
Rd		C57BL/6 J mice, 10 or 30 mg/kg	CCH	BDNF↑ caspase-3, Ac-H3, HDAC2↓	Epigenetic modulation apoptosis	Wan et al. (2017)
		Neuronal Cell, 0.1, 1.0, and 10 μM	OGD/R			
Rd		SD rats, 50 mg/kg	MCAO	NR2B, P- Ser-1303, P-Tyr-1472, P- Ser-1480↓	Neuroprotection	Xie et al. (2016)
		Cortical neurons, 10 μM	OGD			
Rd		SD rats, 10 mg/kg	MCAO	IL-1*α*, IL-1*β*, IL-6, IL-10, IL-18, TNF-*α*, IFN-*γ*, IκB*α*, p65, NF-κB↓	Inflammation	Zhang et al. (2016)
		BV2 cells, 10 μM	OGD			
Rd		SD rats, 30 mg/kg	MCAO	NEIL1, NEIL3 ↑ mtDNA and nDNA damages, caspase-3, TUNEL↓	Attenuate DNA damage, apoptosis	Yang et al. (2016)
Rd		PC12 cells, 0.1, 1, 10, 50 or 100 µm		GAP-43, ERK1/2, AKT↑ MAPK/ERK and PI3K/AKT pathways	Neurite outgrowth, neuronal repair	Wu et al. (2016a)
Rd		SD rats, 1, 2.5, and 5 mg/kg	MCAO	BrdU/DCX, Nestin/GFAP, VEGF, BDNF, pAkt, pERK↑ PI3K/Akt and ERK1/2 pathways	Neurogenesis	Liu et al. (2015)
		PC12 cells, 25, 50, and 100 μmol/L	OGD			
Rd		SD rats, 30 and 10 mg/kg Neuron cells, 10 μM	MCAO	PKB/AKT↑	Attenuates tau protein, reduce sequential cognition impairment	Zhang et al. (2014)
			OGD	ptau, GSK-3*β*↓ PI3K/AKT/GSK-3*β* pathway		
Rd		SD rats, 30 mg/kg	MCAO	GLT-1, p-PKB/Akt, p-ERK1/2↑	Glutamate clearance	Zhang et al. (2013)
		Astrocytes, 10 and 50 μM	OGD	glutamate↓		
Rd		SD rats, 10 mg/kg	MCAO	NF-κB p65, PARP-1 ↓	Inflammation, apoptosis	Hu et al. (2013)
Rd		Hippocampal neurons, 0.1, 1, 10 μM	Glutamate-induced	Ca^2+^ Influx, TUNEL and caspase-3↓	Ca^2+^ Influx	Zhang et al. (2012a)
Rd		SD rats, 10 mg/kg	MCAO	ASIC2a↑	Ca^2+^ Influx	Zhang et al. (2012b)
				TRPM7, ASIC1a↓		
Rd		SD rats, 50 mg/kg	MCAO	ROS, CytoC, AIF↓	Mitochondrial protection, energy restoration, inhibition of apoptosis	Ye et al. (2011d)
Rd		SD rats, 0.1–200 mg/kg	MCAO	iNOS and COX-2↓	Oxidative, inflammatory	Ye et al. (2011c)
Rd		SD rats, 10–50 mg/kg	MCAO	BBB permeability↑	Wider therapeutic window	Ye et al. (2011a)
Rd		C57BL/6 mice, 10–50 mg/kg	MCAO	CAT, SOD2, GPX, GST, GSH/GSSG, complexes I-IV↑ ROS↓	Redox imbalance, oxidative damage, mitochondrial function	Ye et al. (2011b)
Rd		Cortical neurons, 1, 3, 10, 30 and 60 μM	Glutamate-induced	caspase 3, Ca^2+^ influx↓	Apoptosis	Li et al. (2010)
Rd		Hippocampal neurons, 0.1–10 μM	OGD	GPX, SOD, CAT↑ MDA, GSH, GSSG, ROS↓	Oxidative stress	Ye et al. (2009)
Rb1	0.11 ± 0.02%Park et al. (2013)	C57BL/6 mic, 50 mg/kg	dMCAO	GAP43, BDA, cAMP, PKA, pCREB↑ cAMP/PKA/CREB Pathway	Axonal regeneration, motor functional recovery	Gao et al. (2020b)
	0.29–2.0%Chen et al. (2019b)					
Rb1, Rg1		Astrocyte cultures	OGD/R	CAT, complexes I-V, ATP↑	Mitochondrial oxidative	Xu et al. (2019)
		Rb1, 2, 5, 10 µM		ROS↓		
		Rg1, 2, 5, 10 µM				
Rb1, Rh2, Rg1, Rg3, Rg5, Re		PC12 cells Rb1, 50 μg/ml Rh2, 0.5 μg/ml Rg1, 5 μg/ml Rg3, 20 μg/ml Rg5, 100 μg/ml Re, 5 μg/ml	CoCl_2_-induced	ROS, TLR4, MyD88, SIRT1, P65, IL-1*β*, TNF-*α*, IL-6↓	Apoptosis, mitochondrial membrane potential, inflammation	Cheng et al. (2019)
Rb1		SD rats, 50 or 100 mg/kg SH-SY5Y cells, 10 μmol/L	Microperfusion of Glu and CaCl_2_ OGD/R	P-Akt, P-mTOR↑ P-PTEN↓P-AKT/P-mTOR pathway	Neuroprotection, microenvironment	Guo et al. (2018)
Rb1		Wistar rats, 50, 10, 200 mg/kg	MCAO	caspase-3, caspase-9, HMGB1, NF-κB, TNF-*α*, IL-6, NO↓	Apoptosis, inflammation	Liu et al. (2018a)
Rb1		C57BL/6 J mice, 0.5, 1, 5 or 10 mg/kg	MCAO	GSH↑	Antioxidant	Dong et al. (2017)
				MDA, NO, ROS, NOX-1, NOX-4, NADPH, pERK1/2↓		
Rb1		ICR mice, 5, 20 or 40 mg/kg	MCAO	MMP-9, NOX-4 ↓	BBB	Chen et al. (2015)
Rb1		Microglial cell, 100 μg/ml	H_2_O_2_-induced	TNF-*α*, NO, O2-↓	Apoptosis	Ke et al. (2014)
Rb1		SH-SY5Y cells, 1.0, 10 and 100 µM	OGD	p-Akt↑	Aautophagy	Luo et al. (2014)
				LC3II, Beclin1 ↓PI3K/Akt Pathway		
Rb1		SD rats, 100 mg/kg	MCAO	BDNF, GAP-43, NF↑	Neuroprotection	Jiang et al. (2013)
				IL-1, TNF-*α*↓		
Rb1		SD rats, 12.5 mg/kg	MCAO	NF-κB/p65, IKK-*α*, IκB-*α*, TNF-*α*, IL-6↓	Inflammation	Zhu et al. (2012)
Rb1		SD rats, 12.5 mg/kg	MCAO	LC3, Beclin 1↓	Autophagy	Lu et al. (2011)
Rb1		Wistar rats, 40 mg/kg	MCAO	BDNF↑ caspase-3↓	Neurogenesis	Gao et al. (2010)
Rb1		Cynomolgus monkeys, 300 μg/kg	TSM	NeuN↑	Neuroprotection	Yoshikawa et al. (2008)
				TUNEL, GFAP↓		
Rb1		SHR-SP rats, 20 μg/kg	MCAO	VEGF, Bcl-xL↑	Neuroprotection	Sakanaka et al. (2007)
Rb1		Wistar rats, 40 mg/kg	MCAO	GDNF, Bcl-2↑ bax↓	Apoptotic	Yuan et al. (2007)
Rb1		SHR-SP rats, 20 μg/kg	MCAO	Bcl-xL↑	Apoptotic	Zhang et al. (2006)
Rb1		SHR-SP rats, 20 μg/kg	MCAO	Infarcted area↓ scavenging free radicals	neuroprotection	Zhang et al. (1998)
Rb1		Mongolian gerbils, 80 μg/kg	TFI	Hippocampal blood flow↑ scavenging free radicals	neuroprotection	Lim et al. (1997)
Rg1	0.27 ± 0.04%Park et al. (2013)	SD rats, 40 mg/kg	MCAO	Bcl2 ↑	ER, apoptosis	Gu et al. (2020)
	0.32–1.55%Chen et al. (2019b)			Bax, TUNEL, p-PERK, p-eIF2, ATF4↓ PERK-eIF2-*α*-ATF4 signaling pathway		
Rg1		SD rats, 50 mg/kg	MCAO	Glycolysis or gluconeogenesis, amino acid metabolism, lipid metabolism↓	Energy metabolism, amino acids metabolism, lipids metabolism	Gao et al. (2020a)
Rg1		SD rats, 20 mg/kg	tMCAO OGD/R	Nrf2, ARE, HO-1, NQO-1, GCLC, GCLM↑ miR-144 ↓ miR-144/Nrf2/ARE pathway	Oxidative stress	Chu et al. (2019)
		PC12 cells, 0.01–1 μmol/L				
Rg1		SD rats, 10, 20, or 40 mg/kg	MCAO	p-IκB*α*, P65, IL-6, IL-1*β*, TNF-*α*, IFN-*γ*↓	Inflammation	Zheng et al. (2019)
Rg1		C57BL/6 mice,10, 20 or 40 mg/kg hCMEC/D3 cells,0.1–1,000 μM	dMCAO OGD	BrdU+/CD31+, BrdU+/GFAP+, VEGF, HIF-1*α*, p-Akt, p-mTOR↑ PI3K/Akt/mTOR signaling pathway	Angiogenesis	Chen et al. (2019a)
Rg1		C57BL/6 mice, 20, 40 mg/kg	MCAO	BDNF↑ IL-1*β*, TNF-*α*, IL-6, Glu, Asp↓	Neuroprotection	Wang et al. (2018b)
Rg1		SD rats, 6 mg/kg BV2, 8 μg/ml	MCAO OGD	miR-155-5p↓	Neuroprotection	Wang et al. (2018a)
Rg1		SD rats, 30 or 60 mg/kg	MCAO	SOD, CAT, PPAR*γ*↑	Antioxidative, anti-Inflammatory	Li et al. (2017a)
		Cortical neurons, 30 or 60 μM	OGD	MPO, TNF-*α*, IL-6↓		
Rg1		NSCs, 0.01–50 µM	OGD	Bcl-2↑	Apoptosis	Li et al. (2017b)
				Caspase3, Bax, p-p38, p-JNK2↓		
Rg1		SD rats, 40 mg/kg	MCAO	PAR-1↓	BBB permeability	Xie et al. (2015)
Rg1		SD rats, 20, 40 or 60 mg/kg	MCAO	PPAR*γ*, HO-1, bcl-2↑ caspase-3, caspase-9, IL-1*β*, TNF-*α*, HMGB1, RAGE↓ PPAR*γ*/Heme oxygenase-1 (HO-1) signaling	Inflammation, apoptosis	Yang et al. (2015)
Rg1		SD rats, 30, 60 mg/kg	MCAO	Regulate systemic metabolic	Neuroprotection	Lin et al. (2015)
Rg1		Hippocampal neurons, 5, 20, 60 mM	OGD	Calcium influx↓ nNOS↑	Neuroprotection	He et al. (2014)
Rg1		BALB/c mice, 20 or 40 mg/kg	MCAO	mitochondrial membrane potential↑	Apoptosis Ca^2+^ overload	Sun et al. (2014)
		Astrocytes, 10 µM	H_2_O_2_-induced	Ca^2+^, ROS↓		
Rg1		SD rats, 20 mg/kg	MCAO	AQP4↓	BBB	Zhou et al. (2014)
Rg1		PC12 cells, 0.1–10 uM	H_2_O_2_- induced	Akt, ERK1/2 ↑ p-IkB*α*, p-IKK*β*, p65↓ NF-kB pathway	Oxidative stress	Liu et al. (2011)
Rg1		SD rats, 20 mg/kg	MCAO	Ca^2+^↓	Neuroprotection	Zhang et al. (2008b)
		Hippocampal neurons, 110,100 uM	OGD	NMDA receptors and L-type voltage-dependent Ca^2+^ channels		
Rg1		Mongolian gerbils, 5 and 10 mg/kg	MCAO	Brdu↑	Neurogenesis cell proliferation	Shen and Zhang, (2003)
Rg2	0.06 ± 0.04%Park et al. (2013)	SD rats, 2.5, 5 and 10 mg/kg	MCAO	BCL-2, P53↑	Apoptosis	Zhang et al. (2008a)
	0.01–0.09%Chen et al. (2019b)			BAX, HSP70↓		
Rg3	0.05 ± 0.04%Park et al. (2013)	SD rats, 20 mg/kg	MCAO/R	22 differentially expressed miRNAs 415 differentially expressed mRNAs cGMP-PKG, cAMP and MAPK signaling pathways	Neuroprotection	Zhang et al. (2022)
	0.001–0.003%Chen et al. (2019b)					
Rg3		SD rats, 20 mg/kg	MCAO/R	239 differentially expressed lncRNAs 538 differentially expressed mRNAs TNF, NF-κB, cytokine, and other receptor signaling pathways	Neuroprotection	Yang et al. (2022)
Rg3		SH-SY5Y cells, 1, 5, 25, 125 μmol/L	OGD/R	Bcl-2↑	Apoptosis	He et al. (2017)
				Bax, cleaved caspase-3↓		
Rg3		SD rats, 10 and 20 mg/kg	MCAO	calpain I, caspase-3, TUNEL↓	Neuroprotection, apoptosis	He et al. (2012)
Rg3		Mitochondria, 2–16 μM	Ca^2+^, H2O2 induced	ATP, respiratory control ratio ↑	Neuroprotection	Tian et al. (2009)
				MPTP↓		
Rg3		Wistar rats, 10 and 5 mg/kg	MCAO	MDA, ATP ↑	Lipid peroxides, oxidative stress, energy metabolism	Tian et al. (2005)
				SOD, GSH-Px ↓		
Re	0.22 ± 0.03%Park et al. (2013)	SD rats, 5, 10 or 20 mg/kg	MCAO	MDA, H^+^ -ATPase↓ decrease mitochondrial swelling	Oxidative stress	Chen et al. (2008)
	0.44–1.2%Chen et al. (2019b)					
Re		Wistar rats, 5, 10, 20 mg/kg	MCAO	SOD, GSH-Px↑	Oxidative stress	Zhou et al. (2006)
				MDA↓		
CK		PC12 cells, 2, 4, 8 μM	OGD/R	p-mTOR↑p-AMPK, p62, Atg7, Atg5, LC3II/I↓ AMPK-mTOR pathway	Autophagy, apoptosis	Huang et al. (2020)
CK		C57BL/6 mice, 30 mg/kg	MCAO	HO-1↑	Anti-inflammation	Park et al. (2012)
		BV2, 25, 50, 75 μM	LPS	IL-6, MCP-1, MMP-3, and MMP-9↓ ROS, MAPKs, NF-κB/AP-1, and HO-1/ARE signaling pathways		
OA		SD rats, 10, 20 mg/kg	MCAO	Nissl+, NeuN+↑	Antioxidative	Lin et al. (2021)
		SH-SY5Y cells, 10, 20, and 40 µM	OGD/R	GSK-3*β*, HO-1, ROS, TUNEL ↓ GSK-3*β*/HO-1 pathway		
F1		SD rats, 50 mg/kg	MCAO	MVD, IGF-1/IGF1R↑ IGF-1/IGF1R pathway	Angiogenesis, improve focal cerebral blood perfusion	Zhang et al. (2019)
Rh2	0.001–0.006%Chen et al. (2019b)	BV2, 5, 25 μM	LPS and IFN-*γ*-induced	IL-10↑	Inflammation	Bae et al. (2006)
				NO, COX-2, TNF-*α*, IL-1↓ AP-1 and PKA pathway		

KRG, Korean red ginseng; BG, Black ginseng; KGT, Korean ginseng tea; RGE, Red Ginseng Extract; PGE, Panax ginseng extract; GTS, Ginseng total saponins; GTS, Ginseng total saponins; CK, Compound K; OA, Oleanolic acid; HI, Hypoxia-Ischemia; pdMCAO, permanent distal middle cerebral artery occlusion; tMCAO, transient middle cerebral artery occlusion; MCAO/R, middle cerebral artery occlusion/reperfusion; TGCI, transient global cerebral ischemia; TSM,T hromboembolic stroke model; TFI, transient forebrain ischemia; OGD/R, oxygen-glucose deprivation/reoxygenation; CCH, chronic cerebral hypoperfusion; NSCs, Neural stem cells; ASK1, apoptosis signal-regulating kinase 1; NADPH-d, nicotinamide adenine dinucleotide phosphate-diaphorase; TNF-*α*, tumor necrosis factor-*α*; IL-1*β*, interleukin-1 beta; MDA, malondialdehyde; SOD, superoxide dismutase; GPx, glutathione peroxidase; LPO, lipid peroxidation; GSH, glutathione; GR, glutathione reductase; CAT, catalase; GST, glutathione-S-transferase; Ac-H3, acetylated histone H3; HDAC2, histone deacetylase 2; mtDNA, mitochondrial DNA; ROS, reactive oxygen species; ATP, adenosine triphosphate; HMGB1, High-mobility group box 1; MMP-9, matrix metalloproteinase-9; NOX, nicotinamide adenine dinucleotide phosphate oxidase; HSP70, heat shock protein 70; BBB, blood–brain barrier; ER, endoplasmic reticulum stress; MPTP, mitochondrial permeability transition pore; MVD, microvessel density.

**TABLE 2:** Summary of clinical trials of ginsenosides interventions in cerebarl ischemic stroke patients.

Gensinosides	Model	Sample sizes	Inclusion criteria	Evaluaive critera	Results	References
Rd	Acute ischaemic stroke	Ginsenoside Rd group (*n* = 290) placebo group (*n* = 96)	1) 18–75 years of age; 2) had received a clinical diagnosis of primary acute ischaemic stroke and were able to receive the study drug within 72 h after the onset of symptoms; 3) had a score of 5–22 on the NIHSS	NIHSS BI	Ginsenoside Rd improved the NIHSS and mRs scores, and had an acceptable adverse event profile.	Liu et al. (2012)
Rd	Acute ischaemic stroke	Ginsenoside-Rd 10 mg (*n* = 65) ginsenoside-Rd 20 mg (*n* = 67) placebo group (*n* = 67)	1) between 18 and 75 years of age; 2) had a clinical diagnosis of primary acute ischaemic stroke with an onset of the first episode within the previous 72 h; 3) had a score of 5–22 on the NIHSS	NIHSS BI mRs	Ginsenoside Rd improved NIHSS scores at 15 days, no significance of BI and mRs scores at 15 and 90 days.	Liu et al. (2009)

NIHSS, national institutes of health stroke scale; mRs, modified Rankin scalel; BI, barthel index.

### Panax Ginseng and its Neuroprotective Effects

According to the manufacturing processing technique of ginseng, Panax ginseng can be divided into three types: white ginseng, red ginseng, and black ginseng (Hyun et al., 2022). White ginseng is produced by dehydration in the sun without cooking, and red ginseng is steamed at 90–100°C for 2–3 h. Until now, red ginseng is mainly processed in Korea, which is also named Korea red ginseng (KRG). While black ginseng is generated by steaming red ginseng nine times (Jo et al., 2009; Wan et al., 2021). The therapeutic effects of KRG on permanent and transient hypoxic-ischemic brain damage were studied in rats and mice at 100–360 mg/kg per day. In hypoxic-ischemic (HI) mice, 7 days before HI pretreated with KRG, reduced infarct volume, cerebral edema, and degeneration of hippocampal neurons were observed at 6 h, 24 h, 7 days, and 28 days after HI (Liu et al., 2019a; Liu et al., 2020). What’s more, red ginseng pretreatment could also suppress apoptosis in ischemic lesions (Cheon et al., 2013). Recent studies have shown that KRG pretreatment has elicited robust and prolonged anti-oxidative and anti-inflammatory effects after hypoxia-ischemia *via* an Nrf2-dependent manner. While Nrf2-dependent endogenous neuroprotection effects attenuate sensorimotor deficits and gliosis reactive in microglia and astrocytes, they regulate dynamic glutamine synthetase (GS) and aquaporin 4 (AQP4) expressions, thus improving long-term functional recovery (Liu L. et al., 2018; Liu et al., 2019b; Liu et al., 2020). Red ginseng could play the effect of anti-oxidant by reducing the level of lipid peroxidation (Ban et al., 2012), and increasing the expression of GSH, CAT, GST, glutathione peroxidase GPx and SOD (Kim et al., 2009) (Shah et al., 2005). Meanwhile, the neuroprotection of anti-inflammation may raise IL-10 expression and reduce the levels of TNF-*α*, IL-1*β*, and IL-6 in serum (Lee et al., 2011). In addition, black ginseng is helpful for the treatment of vascular dementia *via* reduced loss of cholinergic immunoreactivity and nicotinamide adenine dinucleotide phosphate-diaphorase (NADPH-d)-positive neurons in the hippocampus (Park H. J. et al., 2011). Ginseng total saponins could increase the number of BrdU+/NeuN+ cells to induce endogenous neural stem cell activation (Zheng et al., 2011), further supporting the beneficial role of ginseng in ischemic stroke.

### Ginsenoside Rd and its Neuroprotective Effect

Ginsenoside Rd is one of the major ginsenosides responsible for pharmaceutical activities and has been demonstrated to exert significant neuroprotective in preclinical and clinical studies. *In vitro* and *in vivo* studies show that ginsenoside Rd could improve neuron survival and decrease neuron apoptosis. Ginsenoside Rd modulates the balance between acetylated histone H3 (Ac-H3) and histone deacetylase [histone deacetylase 2 (HDAC2)], thus upregulating BDNF in chronic cerebral hypoperfusion (CCH) mice and OGD/R neurons (Wan et al., 2017). Ginsenoside Rd significantly inhibits glutamate-induced Ca^2+^ entry in cortical neurons and prevents cell apoptosis (Li et al., 2010). A recent study shows that ginsenoside Rd regulates cerebral ischemia/reperfusion injury by exerting an anti-pyroptotic effect through the miR-139-5p/FoxO1/Keap1/Nrf2 axis (Yao et al., 2022). What’s more, ginsenoside Rd administration could enhance ischemic stroke-induced cognitive impairment and downregulate tau protein phosphorylation *via* the PI3K/AKT/GSK-3*β* pathway (Zhang et al., 2014). Glutamate is essential for excitatory synapse transmission; however, overstimulation of ionic glutamate receptors can trigger excessive calcium influx, leading to excitotoxicity of neurons. Ginsenoside Rd protects neurons against glutamate-induced excitotoxicity by inhibiting Ca^2+^ influx (Zhang C. et al., 2012), attenuating the expression of transient receptor potential melastatin 7 (TRPM7) and acid-sensing ion channels 1a (ASIC1a) (Zhang Y. et al., 2012), and mitigating DAPK1-mediated NR2b phosphorylation and attenuating calcineurin activity (Xie et al., 2016; Zhang C. et al., 2020). Ginsenoside Rd administration promotes glutamate clearance by upregulating the expression of glial glutamate transporter-1 (GLT-1) through PI3K/AKT and ERK1/2 pathways (Zhang et al., 2013).

Pretreatment of ginsenoside Rd plays antiapoptotic and anti-inflammatory effects in MCAO rats through inhibiting poly (ADP-ribose) polymerase-1, preventing the mitochondrial release of apoptosis-inducing factor (AIF), and reducing the accumulation of NF-κB p65 subunit nuclear (Hu et al., 2013). Another study showed that ginsenoside Rd could eliminate inflammatory injury by inhibiting the expression of iNOS and cyclooxygenase-2 (COX-2) (Ye et al., 2011c). Oxidative stress caused by ischemic stroke leads to DNA damage and triggers cell death. Ginsenoside Rd could upregulate the endogenous antioxidant system, preserve the mitochondrial respiratory chain complex and aconitase activities, downregulate mitochondrial hydrogen peroxide production, and stabilize mitochondrial membrane potential (Ye et al., 2009; Ye et al., 2011b; Yang et al., 2016). Another similar report showed that ginsenoside Rd minimizes mitochondria-mediated apoptosis following focal ischemia by reducing the mitochondrial release of cytochrome c (CytoC) and AIF. *In vitro* studies further exhibited that ginsenoside Rd could attenuate mitochondrial swelling, preserve MMP, and decrease ROS production (Ye et al., 2011d). Following ischemic stroke, impaired cell volume regulation can lead to cytotoxic cell swelling, disruption of BBB integrity, and brain edema. Ginsenoside Rd could pass through the intact BBB and exert neuroprotection effects in transient and permanent MCAO rat models (Ye et al., 2011a). In addition, ginsenoside Rd attenuates BBB by inhibiting proteasome activity and sequentially suppressing the NF-κB/MMP-9 pathway (Zhang X. et al., 2020). At the same time, ginsenoside Rd could promote neurogenesis *via* upregulating the expression of VEGF, BDNF, and growth-associated protein of 43 kDa (GAP-43) and activating the PI3K/Akt and ERK1/2 dependent pathways (Liu et al., 2015; Wu S. D. et al., 2016).

Two randomized, double-blind, placebo-controlled, phase II multicenter clinical trials involving 199 patients (Liu et al., 2009) and 390 patients (Liu et al., 2012) with acute ischemic stroke showed that Rd could improve patients’ neurologic deficits scores at 15 or 90 days and ameliorate disability by modified Rankin Scale (mRS) score at 90 days after stroke. The therapeutic effect of ginsenoside Rd may be related to its capability to suppress microglial proteasome and secondary inflammation (Zhang et al., 2016). The studies suggested that ginsenoside Rd is a promising neuroprotectant in acute ischemic patients.

### Ginsenoside Rb1 and its Neuroprotective Effects

Ginsenoside Rb1 is one of the main bioactive saponins in ginseng, which could alleviate cerebral ischemia injury *via* modulating apoptosis, autophagy, oxidative, inflammation, BBB permeability, and promoting neurogenesis (Jiang et al., 2013; Luo et al., 2014; Chen et al., 2015; Cheng et al., 2019). Apoptotic caspases further classified as initiator caspases (Caspase-8, -9, -10), and effector caspases (Caspase-3, -6, -7) based on their functions (Wu Y. et al., 2016). Ginsenoside Rb1 could inhibit apoptosis and attenuate damaged neurons by downregulation of the expression of caspase-3, caspase-9 (Liu A. et al., 2018), nitric oxide, and superoxide (Ke et al., 2014), and up-regulating the expression of the mitochondrion associated antiapoptotic factor Bcl-xL (Zhang et al., 2006). Ginsenoside Rb1 could inhibit the expression of Beclin-1 and LC3-II *via* activation of PI3K/Akt pathway (Lu et al., 2011; Luo et al., 2014). The neuroprotective effect of ginsenoside Rb1 is also related to the activation of Akt/mTOR signaling pathway and inhibition of P-PTEN protein (Guo et al., 2018).

Free radicals can be excessively produced following cerebral ischemia. Ginsenoside Rbl protects the cerebral cortex and hippocampal CA1 neurons against ischemic damage by scavenging free radicals (Lim et al., 1997; Zhang et al., 1998). Administration of Rb1 or Rg1 could improve the mitochondrial and reduce ROS production in OGD/R cultured astrocytes, with increased activity of CAT, complexes I, II, III, and V, elevated level of mtDNA and ATP, and attenuated the MMP depolarization (Xu et al., 2019). Furthermore, ginsenoside Rb1 also showed an antioxidative effect in aged mice (Dong et al., 2017). Inflammation plays an important role in the pathophysiological process after ischemic stroke, which could induce secondary brain damage (Rajkovic et al., 2018). Ginsenoside Rbl could exert anti-inflammatory effects by downregulating the expression of IL-6, and TNF-*α* (Zhu et al., 2012), which is associated with TLR4/MyD88 and SIRT1 signaling pathways (Cheng et al., 2019). High mobility group box1 (HMGB1) is a highly abundant non-histone DNA-binding nuclear protein and is a crucial pro-inflammatory factor in ischemic stroke. Administration of Ginsenoside Rb1 could also attenuate cerebral ischemic reperfusion-induced apoptosis and inflammation *via* inhibiting HMGB1inflammatory signals (Liu A. et al., 2018). In addition, ginsenoside Rb1 protects BBB integrity following cerebral ischemia and reduces brain edema by suppressing neuroinflammation induction of MMP-9 and NOX4-derived free radicals (Chen et al., 2015). Ginsenoside Rb1 has a positive effect on neurogenesis, probably *via* improving the expression of NeuN, BDNF, glial-derived neurotrophic factor (GDNF), and growth-associated protein 43 (GAP43), while decreasing the expression of TUNEL, caspase-3, and GFAP (Yuan et al., 2007; Yoshikawa et al., 2008) (Jiang et al., 2013) (Gao et al., 2010). Intravenous infusion of ginsenoside Rb1 prevents ischemic brain damage through upregulation of VEGF and Bcl-xL (Sakanaka et al., 2007). In addition, ginsenoside Rb1could promote functional motor recovery in post-stroke mice by stimulating axonal regeneration and brain repair by regulating the cAMP/PKA/CREB pathway (Gao X. et al., 2020).

### Ginsenosides Rg1, Rg2, Rg3, Rg5 and Their Neuroprotective Effects

Ginsenoside Rg, including Rg1, Rg2, Rg3, and Rg5, has been widely used in cerebral ischemic stroke with therapeutic effects of anti-apoptosis (Zhang G. et al., 2008), antioxidant (Li et al., 2017a), anti-inflammation (Zheng et al., 2019), regulating energy metabolism (Gao J. et al., 2020), and promoting angiogenesis (Chen J. et al., 2019) and neurogenesis (Shen and Zhang, 2003).

Ginsenoside Rg1 could reduce the neurological deficit scores, brain edema, and infarct volume in MCAO mice and inhibit intracellular Ca^2+^ overload and ROS production in astrocytes (Sun et al., 2014). Neuron apoptosis, inflammation, and oxidative stress are the main pathological characteristics of cerebral ischemia stroke. Ginsenoside Rg1 protects NSCs from OGD-induced cell apoptosis and oxidative stress *via* inhibiting the phosphorylation of p38/JNK2 (Li et al., 2017b), while Rg1 combined with mannitol protects neurons against apoptosis through the PERK-eIF2-*α*-ATF4 signaling pathway (Gu et al., 2020). PPAR*γ*/Heme oxygenase-1 (HO-1) signaling was critical in mediating apoptosis and inflammation, while ginsenoside Rg1 could activate PPAR*γ*/HO-1 and provide neuroprotective effects *via* modulating the expression of levels of PPAR*γ*, Bcl-2, cleaved caspase-3, cleaved caspase-9, IL-1*β*, TNF-*α*, HMGB1 (Yang et al., 2015). Similarly, ginsenoside Rg2 and Rg3 exert a neuroprotective effect against apoptosis by decreasing the levels of Bax, and increasing the levels of Bcl-2 (He et al., 2012; He et al., 2017) ^[86]^. Ginsenoside Rg1, Rg3, Rg5, Rb1, Rh2, and Re could reduce cerebral ischemic damage by inhibiting NF-κB transcriptional activity and the expression of pro-inflammatory cytokines (Cheng et al., 2019; Zheng et al., 2019). Administration of ginsenoside Rg1 in combination with geniposide protected against focal cerebral ischemia injury *via* microglial microRNA-155-5p inhibition (Wang J. et al., 2018). Moreover, the neuroprotection of ginsenoside Rg3 against ischemic injury is associated with multiple lncRNAs, miRNAs sand mRNAs, which mainly related to the tumor necrosis factor (TNF), NF-κB, cytokine, and cGMP-PKG, cAMP and MAPK signaling pathways (Yang et al., 2022; Zhang et al., 2022).

A previous study confirmed that ginsenoside Rg1 exerts the neuroprotective effect of antioxidant *via* downregulation of the NF-kB signaling pathway, and activation of Akt and ERK1/2 in H_2_O_2_-induced cell injury (Liu et al., 2011). *In vitro* and *in vivo* studies showed that Ginsenoside Rg1 significantly increased PPAR*γ* expression and regulated the oxidative stress and inflammation after ischemic injury (Li et al., 2017a). Additionally, ginsenoside Rg1 could alleviate oxidative stress *via* inhibiting miR-144 and promoting the Nrf2/ARE pathway after ischemic/reperfusion injury (Chu et al., 2019). What’s more, ginsenoside Rg1 exerts neuroprotective effects by blocking the intracellular calcium overload and decreasing the concentration of free calcium and iNOS activity after OGD exposure (He et al., 2014), the inhibition of calcium influx *via* NMDA receptors and L-type voltage-dependent Ca^2+^ channels (Zhang Y. F. et al., 2008). Metabolic changes play an important role in cerebral ischemic damage. The potential therapeutic effect of ginsenoside Rg1 is possible *via* suppressing the systemic metabolic changes in cerebral injury rats (Lin et al., 2015). NSCs transplantation combined with ginsenoside Rg1 could significantly improve the cerebral infarct and neurological deficits *via* intervening energy metabolism, amino acids metabolism, and lipids metabolism (Gao J. et al., 2020). Besides, ginsenoside Rg3 could decrease the activities of SOD and GSH-Px, and enhance MDA and ATP levels after cerebral ischemia, which provide neuroprotection *via* reducing lipid peroxides, scavenging free radicals, and improving mitochondrial energy metabolism (Tian et al., 2005; Tian et al., 2009).

Angiogenesis plays a crucial role in reconstructing brain tissue and recovering neurological function after an ischemic stroke. Ginsenoside Rg1 could promote cerebral angiogenesis through the PI3K/Akt/mTOR signaling pathway, *via* upregulating the expressions of VEGF, HIF-1*α*, PI3K, p-Akt, and p-mTOR, and significantly increase the proliferation, migration and tube formation of endothelial cells (Chen J. et al., 2019). Besides, ginsenoside Rg1 exerts neuroprotection in cerebral ischemic injury *via* increasing the expression of BDNF in the hippocampal CA1 region and decreasing the expression of IL-1*β*, IL-6, and TNF-*α* in serum (Wang L. et al., 2018), as well as promoting the neurogenesis in the dentate gyrus of gerbils after global ischemia (Shen and Zhang, 2003). Ginsenoside Rg1 could also ameliorate neurological injury by attenuating BBB permeability, which is related to the downregulation of PAR-1 and aquaporin 4 expressions (Xie et al., 2015) (Zhou et al., 2014).

### Other Ginsenosides and Their Neuroprotective Effects

In addition to ginsenosides summarized above, ginsenoside Re, Rh2, F1, and Compound K, Oleanolic acid may also play a neuroprotective role in treating cerebral ischemic stroke. Ginsenoside Re significantly improved mitochondrial membrane fluidity and decreased mitochondrial swelling, which ameliorated lipid peroxidation and protected neurons *via* improving the activities of SOD and GSH-Px, and reducing the content of MDA in the rat brain (Zhou et al., 2006; Chen et al., 2008). Oleanolic acid (OA) exerts neuroprotective effects *via* reducing ROS production and suppressing the activation of GSK-3*β*, and upregulating the expression of HO-1throgh GSK-3*β*/HO-1 signaling pathway in OGD/R induced SH-SY5Y cells and MCAO rats (Lin et al., 2021). Compound K (CK), a ginseng saponin metabolite, showed the neuroprotective effect of anti-inflammatory *via* suppressing microglial activation through inhibiting ROS, MAPK, and NF-κB/activator protein-1 (AP-1) and enhancement of HO-1 signaling (Park et al., 2012). Pretreatment of CK protects against neuron damage by increasing cell viability and decreasing ROS generation, mitochondrial damage, and Ca^2+^ overload. What’s more, OGD/R-induced autophagy and apoptosis in neurons could be regulated by modulating the AMP-activated protein kinase (AMPK) and mTOR pathway (Huang et al., 2020). Ginsenoside Rh2 inhibited the expression of COX-2, TNF-*α*, and IL-1*β*, and promoted the anti-inflammatory cytokine IL-10, depending on the AP-1 and protein kinase A (PKA) pathway, which is more potent than the anti-inflammatory effect of ginsenoside Rg3 (Bae et al., 2006). Ginsenoside F1 could promote angiogenesis through the insulin-like growth factor 1 (IGF-1)/insulin-like growth factor 1 receptor (IGF1R) pathway and might also enhance focal cerebral blood perfusion and increase cerebral microvessel density in MCAO rats (Zhang et al., 2019).

## Conclusion and Perspectives

**FIGURE 2:**
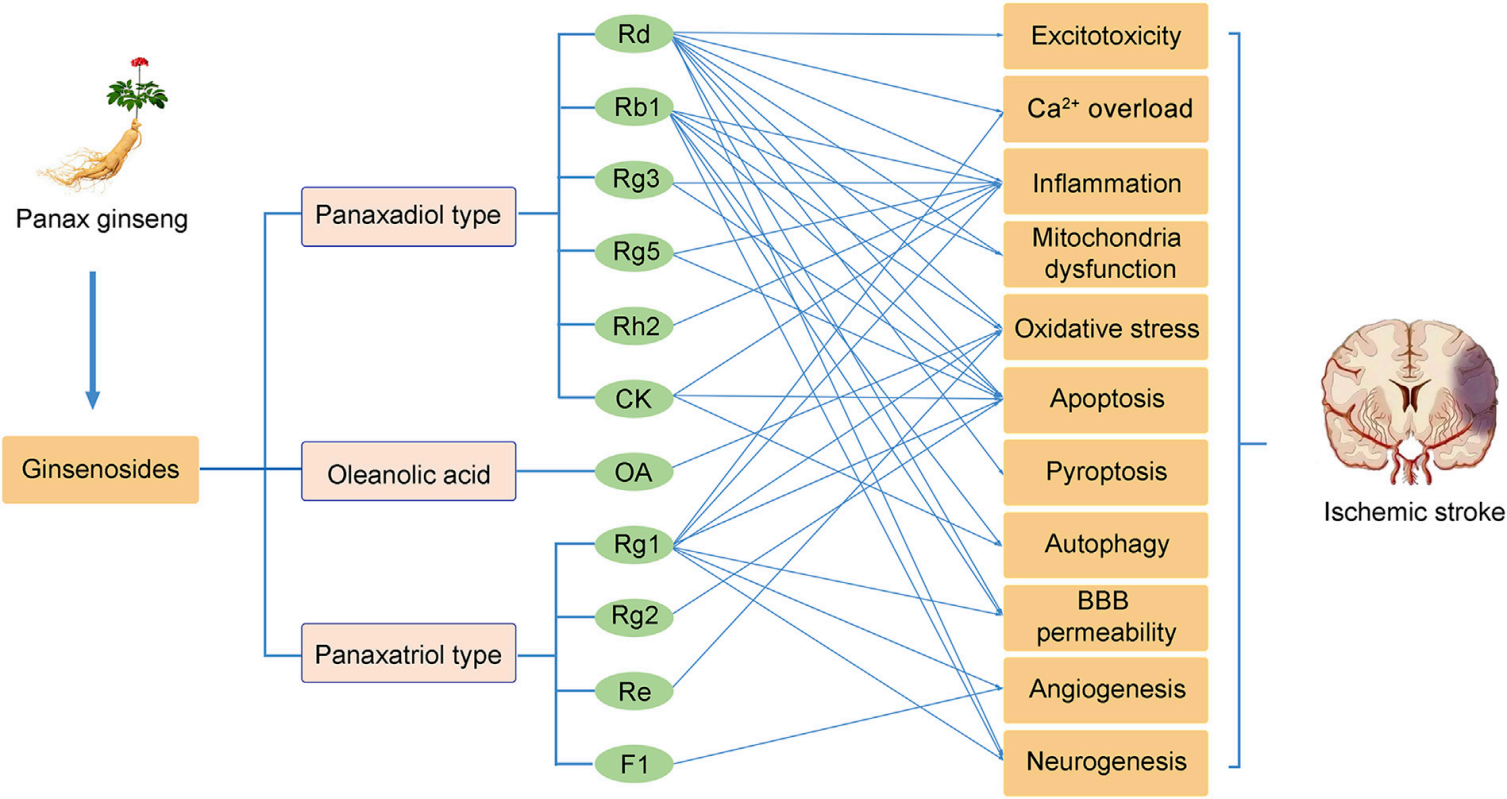
The neuroprotective effects of ginsenosides on cerebral ischemic stroke.

**FIGURE 3:**
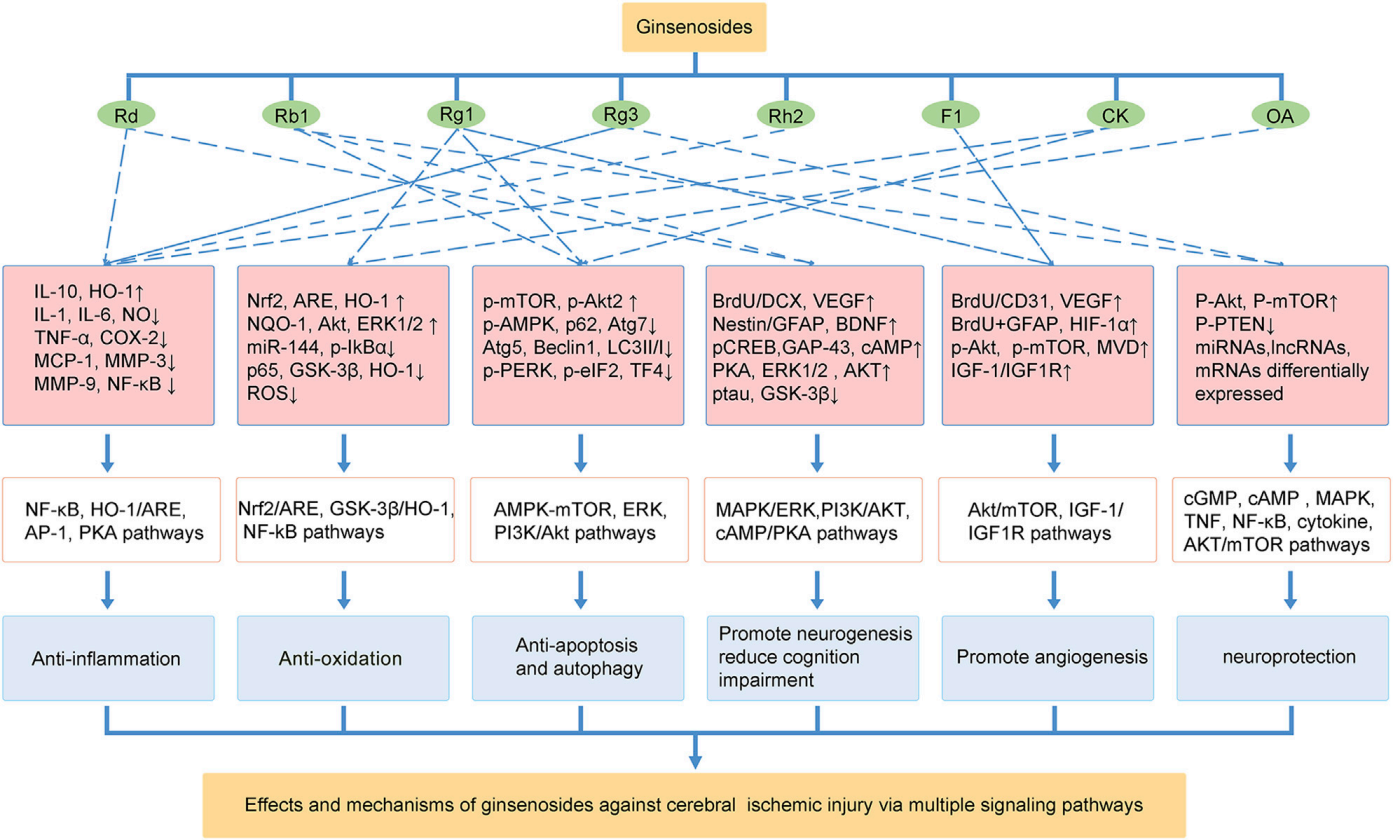
The effects and mechanisms of ginsenosides against cerebral ischemic injury *via* multiple signaling pathways.

Currently, effective therapies for preventing and treating patients with ischemic stroke remain a challenge. Panax ginseng has been widely used in eastern countries for various diseases. The neuroprotective effects of ginseng or ginsenosides on preclinical and clinical ischemic stroke injury have been demonstrated during the last decade. This review concludes our recent findings related to the effects of ginseng and ginsenosides against ischemic stroke. As shown in Figure 2, ginsenoside Rd, Rb1 and Rg1 are the most commonly used in treating ischemic stroke. Mechanisms underlying the neuroprotective effects of ginseng or ginsenosides include regulation of excitotoxicity, Ca^2+^ overload, inflammation, mitochondria dysfunction, oxidative stress, apoptosis, pyroptosis, autophagy, BBB permeability, improving angiogenesis and neurogenesis. These effects can potentially improve abnormal neurobehaviors, such as sensorimotor or cognitive deficits. Ginseng and ginsenosides exert neuroprotective effects *via* modulating multiple signaling pathways, such as MAPK/ERK, PI3K/AKT, cAMP/PKA, AMPK/mTOR, NF-κB, Nrf2, GSK-3*β*/HO-1, IGF-1/IGF1R pathways, to block the pathological damage of neurons and promote the neural remodeling of stroke (Figure 3).

Although numerous preclinical studies have been conducted on the neuroprotective effects of ginseng and ginsenosides in the treatment of ischemic stroke, there are few clinical trials of ginsenosides in treating ischemic stroke. Thus, further high-quality studies are needed to establish the clinical efficacy of ginsenosides. In addition, most experimental stroke models were induced by MCAO in young rats or mice, and only a few aged animals or models with diabetes were used, while the clinical patients are more likely to be associated with hypertension, hyperlipidemia, hyperglycemia, or other diseases. Therefore, it is necessary to study the neuroprotective effects of Panax ginseng or ginsenosides against ischemic stroke with pseudo-clinical models, which will provide a reliable basis for the clinical application of ginseng. Overall, this review describes the recent progress of pharmacological research on ginseng and ginsenosides in ischemic stroke and points out the issues that future research should focus on, which is of great importance for understanding the use of ginseng in the prevention and treatment of ischemic stroke.
